# Evaluation of Risk Factors for Lung Cancer Among Never Smokers and Their Association With Common Driver Mutations

**DOI:** 10.7759/cureus.56024

**Published:** 2024-03-12

**Authors:** Rohit Shirgaonkar, Prasanta R Mohapatra, Manoj K Panigrahi, Pritinanda Mishra, Sourin Bhuniya, Subho Sarkar, Aswathy Girija, Afshan Shaik, Swadesh Mohanty, Akshaya Moorthy

**Affiliations:** 1 Pulmonary Medicine and Critical Care, All India Institute of Medical Sciences, Bhubaneswar, Bhubaneswar, IND; 2 Pathology, All India Institute of Medical Sciences, Bhubaneswar, Bhubaneswar, IND; 3 Pulmonary Medicine, All India Institute of Medical Sciences, Bhubaneswar, Bhubaneswar, IND

**Keywords:** mutations, etiology, risk factors, non-smokers, lung cancer

## Abstract

Introduction: The majority of lung cancers are caused by tobacco use, which is linked to lung tumors of all major histological types. A considerable fraction of lung cancer cases, the vast majority of which are adenocarcinomas, occur in "never smokers," who are characterized as having smoked fewer than 100 cigarettes in their lives. The primary objective was to assess risk factors for lung cancer in non-smokers. In contrast, secondary objectives included evaluating histological subtype, staging, and performance status and exploring associations between risk factors and common driver mutations.

Material and methods: The study was a single-center, observational, case-control study done at All India Institute of Medical Science, Bhubaneswar, India that focused on non-smokers with lung cancer. It included 145 cases and 297 controls, with statistical analyses such as chi-square tests and logistic regression used to assess associations between risk factors and lung cancer, considering factors such as socioeconomic status, body mass index (BMI), occupation, outdoor and indoor air pollution, personal habits, and medical history.

Results: The study, comprising 145 lung cancer cases in non-smokers and 297 controls, found that 92.4% (134/145) of cases had adenocarcinoma, 6.9% (10/145) had squamous cell carcinoma, and 0.7% (1/145) had small cell carcinoma. Significant associations were observed for high-risk occupations, indoor biomass use without proper ventilation, low BMI, and family history of lung cancer. Specific pre-existing lung conditions like old pulmonary tuberculosis and asthma were linked to increased and decreased odds of developing lung cancer, respectively. Environmental factors, living near heavy industry, and dietary habits showed significant associations. A significant association was not found between the driver mutations and the risk factors studied.

Conclusion: This single-center study sheds light on significant risk factors influencing lung cancer development among non-smokers. The predominant occurrence of adenocarcinoma and associations with high-risk occupations, indoor biomass exposure, low BMI, and family history emphasize the multifaceted nature of non-smoking-related lung cancer. The findings underscore the importance of comprehensive risk assessment and targeted preventive strategies in this population.

## Introduction

According to the International Agency for Research on Cancer GLOBOCAN 2020, Lung cancer was one of the leading causes of cancer death, with an anticipated 1.8 million deaths (18%) in 2020 [1]. Lung cancer accounts for 10.6% of the total disease burden in India. There were an estimated 1,03,371 cases of lung cancer in 2022. As per the Indian Council of Medical Research report of 2025 projections, the number of lung cancer cases shall continue to rise in males and females [2]. Lung cancer among non-smokers is affected by several factors, including genetic susceptibility, poor diet, occupational exposure, and air pollution [3]. Up to 10-20% of lung cancers have been attributed to occupational exposures [4].

Socioeconomic disparities, particularly the worse economic conditions, account for higher incidence and mortality from lung cancer in rural communities [5]. Many of the chemicals in wood smoke are carcinogenic and have well-documented adverse effects on human health [6]. Fruits and vegetable consumption, rich in antioxidants, can reduce the chance of developing cancer [7]. Paradoxically, a higher body mass index (BMI) is linked to reduced lung cancer risk and improved outcomes, implicating complex mechanisms [8]. Hormonal replacement therapy, particularly estrogen-induced cell proliferation, is evaluated for its role in lung cancer pathogenesis [9].

There is evidence in non-smokers of increased lung cancer risk, however, with varying conclusions. This study aims to gain a deeper understanding of the factors contributing to this risk among non-smokers. Therefore, we have made efforts to establish stronger evidence for causal relationships and evaluate the association of lung cancer in those who have never smoked. Furthermore, this knowledge will add to planning strategies for early detection, screening, and prevention of the disease.

## Materials and methods

The study was conducted at All India Institute of Medical Science, Bhubaneswar's pulmonary medicine outpatient and inpatient department, using a single-center, observational, case-control approach. The study's goal was to look at the risk factors for lung cancer in non-smokers. The study was conducted from January 2022 to July 2023. The study included 442 participants, 145 cases, and 297 controls. Patients diagnosed with lung cancer who are never smokers (never smoked/smoked less than 100 cigarettes in their lifetime) admitted to pulmonary medicine were included as "cases." Other patients who are never smokers with no symptoms or signs, normal chest radiographs, and also accompanying attendants of patients who are asymptomatic and nonsmokers were taken as "control." Institutional Ethics Committee - All India Institute of Medical Science, Bhubaneswar issued approval 2021-22/85.

Statistical analysis

Chi-square tests and bivariate and multivariable binary logistic regression analyses were employed to evaluate the association between predictor and outcome variables. For multivariable analysis, exposure variables (except for the subgroup analysis of the high-risk occupation) with a p-value <0.1 in simple binary logistic regression were included. A significance level of 0.05 and a 95% confidence interval were used for both crude odds ratios (COR) and adjusted odds ratios (AOR). Participants were matched for age, sex, and address in a 1:2 ratio (one case matched with two controls).

Data collection

Participants' information, including name, age, gender, and permanent address, was recorded after written consent was obtained. Address data aided in categorizing Odisha and West Bengal into geographical regions, districts, and zones i.e., Medinipur East and West, Central North and South Odisha. Participants were divided into urban and rural groups, and heavy industry inquiries were conducted within 3 km of their homes.

Socio-economic status (SES) and BMI

The BG Prasad scale for the year 2021 was used to calculate the socio-economic status [10]. BMI was calculated in kg/m^2^, and nutritional status was decided based on the Asian criteria of BMI cut-off.

Occupation

In a paper by Ahrens et al. on the analysis of occupational lung cancer, occupations were divided into known (list A) and suspected (list B) based on lung cancer association [11]. In our study, those participants who belonged to lists A and B were classified as high risk for lung cancer. Those employed and those who did not belong to the list were unemployed, and housemakers were classified as low risk. In the high-risk group, the duration of work and the use of respiratory protective equipment were evaluated.

Outdoor air pollution

The PM 2.5 and Air Quality Index (AQI) assessed exposure to ambient air pollution depending on the residential address. The average exposure for the year preceding the presentation was considered, with AQI and PM 2.5 levels categorized into categories.

Indoor air pollution

Participants were asked about their biomass fuel use, including the type of biomass and location. The length of exposure and the use of kerosene stoves were documented. Tobacco smoke exposure in the home and workplace was investigated.

Personal factors

Smokeless tobacco use and alcohol intake were queried. Participants were asked about their daily vegetable intake. The participants were questioned for pre-existing lung diseases, and a family history of lung cancer was assessed. If the family member with lung cancer smoked, a family history of lung cancer was taken as unfavorable for the participant.

Additional factors

Female participants were asked about their use of oral contraceptives. Lung cancer cases were classified based on type, mutations (epidermal growth factor receptor (EGFR), anaplastic lymphoma kinase (ALK), ROS 1 (ROS proto-oncogene 1, receptor tyrosine kinase)), and disease stage upon presentation. The study also examined the association between driver mutations and age, gender, domicile, SES, BMI, occupation, smokeless tobacco use, alcohol intake, dietary factors, pre-existing lung illness, family history, and oral contraceptive use.

## Results

The study delved into various factors influencing lung cancer in non-smokers, encompassing demographic, occupational, environmental, and health-related aspects. The p-value obtained on the chi-square test for the studied variables is presented in Table 1.

**Table 1:** The "p-value" obtained using the chi-square test for various studied variables High-risk jobs: Chemists, farmers involved in farming activities like plowing, harvesting, manuring, and weeding with direct spraying of pesticides, mechanics, production unit of vinyl sheet, construction workers, welding industry employees, electricians, coal mine workers, workers in the furniture industry, carpenter, workers in brass factory, iron factory, plumber, steel manufacturing plant, and rubber industry. Low-risk jobs: Teachers, farmers involved in farming activities like plowing, harvesting, manuring weeding without direct spraying of pesticides, cloth merchants, jewelry shop owners, mobile phone technicians, tailors, grocery store owners, fruit vendors, priests, computer technicians, tea vendor, workers in a botanical garden, fisherman, security guard, domestic helper, retired revenue inspector, army personnel, police inspector, railway employee, financial advisor, the worker in a botanical plant, housemaker and unemployed Use of biomass, types of fuel, duration of biomass, ventilation in biomass place, and use of kerosene stove assessed indoor air pollution at home. Air Quality Index and PM 2.5 were used to assess the outdoor air quality depending on the residential address.

Variable	Cases (%) (n=145)	Controls (%) (n=297)	Chi-square (p-value)
Age group			
- 21-30 years	06 (4.1)	08 (2.7)	0.166
- 31-40 years	08 (5.5)	41 (13.8)	
- 41-50 years	31 (21.4)	69 (23.2)	
- 51-60 years	54 (37.2)	94 (31.6)	
- 61-70 years	32 (22)	63 (21.21)	
- 71-80 years	14 (9.6)	22 (7.4)	
Sex			
- Male	86 (59.3)	178 (59.9)	0.90
- Female	59 (40.7)	119 (40.1)	
Place of stay			
- Odisha	89 (61.4)	184 (62.0)	0.907
- West Bengal	56 (38.6)	113 (38.0)	
Residence			
- Rural	25 (17.2)	42 (14.1)	0.394
- Urban	120 (82.8)	255 (85.9)	
Place of residence			
- Central Odisha	72 (49.7)	156 (52.5)	0.896
- Southern Odisha	9 (6.2)	15 (5.1)	
- Northern Odisha	8 (5.5)	13 (4.4)	
- Medinipur East	39 (26.9)	84 (28.3)	
- Medinipur West	17 (11.7)	29 (9.8)	
Socioeconomic status			
- Upper and upper middle	50 (34.5)	92 (31)	0.006
- Middle-class	29 (20)	102 (34.3)	
- Lower and lower middle	66 (45.5)	103 (34.7)	
Occupation			
- Low risk	68 (46.9)	213(71.7)	<0.001
- High risk	77 (53.1)	84 (28.3)	
Duration of occupation			
(High risk) (n=161)			
- 1-10 years	3 (3.9)	5 (6)	0.037
- 11-20 years	12(15.6)	19 (22.6)	
- 21-30 years	27 (35.1)	40 (47.6)	
- >30 years	35 (45.5)	20 (23.8)	
Use of protective equipment			
(High risk) (n=161)			
- Yes	9 (11.7)	21 (25)	0.030
- No	68 (88.3)	63 (75)	
Use of biomass fuel			
- Yes	117 (80.7)	221 (74.4)	0.144
- No	28 (19.3)	76 (25.6)	
Types of fuel used			
- Agricultural residue	60 (51.3)	93 (42.1)	0.139
- Animal residue	19 (16.2)	41 (18.6)	
- Forest residue	29 (24.8)	77 (34.8)	
- Solid and sewage waste	9 (7.7)	10 (4.5)	
Duration of biomass use (hours/day)			
- Less than 5 hours	47 (40.2)	120 (54.3)	0.013
- More than 5 hours	70 (59.8)	101 (45.7)	
Ventilation in biomass place			
- Indoor ventilated	35 (29.9)	144 (65.2)	0.001
- Indoor non-ventilated	55 (47.0)	13 (5.9)	
- Outdoor	27 (23.1)	64 (29.0)	
Use of kerosene stove			
- Yes	19 (13.1)	42 (14.1)	0.766
- No	126 (86.9)	255 (85.9)	
Industries/factories around <3 km of the residential area			
- Yes	8 (5.5)	5 (1.7)	0.025
- No	137 (94.5)	292 (98.3)	
PM 2.5 levels			
- Good	8 (5.5)	9 (3.0)	0.365
- Moderate	107 (73.8)	217 (73.1)	
- Unhealthy	30 (20.7)	71 (23.9)	
Air Quality Index			
- Good/satisfactory (<100)	113 (77.9)	240 (80.8)	0.479
- Moderate (100-200)	32 (22.1)	57 (19.2)	
Consumption of vegetables			
- <2 times/ week	46 (31.7)	32 (10.8)	<0.001
- 3-5 times/ week	90 (62.1)	141 (46.5)	
- >5 times/week	9 (6.2)	124 (41.8)	
Body mass index			
- Healthy weight	71 (49)	171 (57.6)	<0.001
- Low BMI	65 (44.8)	53 (17.8)	
- Overweight and obese	9 (6.2)	73 (24.6)	
Pre-existing lung disease			
- Yes	44 (30.3)	87 (29.3)	0.082
- No	101 (69.7)	210 (70.7)	
Type of lung disease			
- Pulmonary tuberculosis	23 (52.3)	19 (21.8)	0.002
- Bronchiectasis	2 (4.5)	3 (3.4)	
- Asthma	8 (18.2)	47 (54.0)	
- Interstitial lung disease	5 (11.4)	5 (5.7)	
- COPD	6 (13.6)	13 (14.9)	
Family history of lung cancer			
- Yes	9 (6.2)	6 (2.0)	0.047
- No	136 (93.8)	291 (98.0)	
Exposure to environmental tobacco smoke			
- Yes	21 (14.5)	36 (12.1)	0.487
- No	124 (85.5)	261 (87.9)	
Smokeless tobacco			
- Yes	22 (15.2)	63 (21.2)	0.130
- No	123 (84.8)	234 (78.8)	
Use of oral contraceptive pills			
- Yes	22 (37.3)	36 (30.3)	0.346
- No	37 (62.7)	83 (69.7)	
Alcohol consumption			
- Yes	12 (8.3)	14 (4.7)	0.198
- No	133 (91.7)	283 (95.3)	

The bivariate and multivariate regression analysis results of the significant variables are shown in Table 2.

**Table 2:** The bivariate and multivariate regression analysis results of the significant variables COR: Crude odds ratios; TB: Tuberculosis; AOR: Adjusted odds ratios; COPD: Chronic obstructive pulmonary disease

Variables	Cases (%) (n=145)	Controls (%) (n=297)	COR (95% CI) (Bivariate)	P-value (Bivariate)	AOR (95% CI) (Multivariable)	P-value (Multivariable)
Socioeconomic status						
Upper and upper middle	50 (34.5)	92 (31)	Ref		Ref	
Middle-class	29 (20)	102 (34.3)	0.52 (0.30-0.89)	0.018	0.355 (0.143-0.881)	0.025
Lower and lower middle	66 (45.5)	103 (34.7)	1.189 (0.74-1.87)	0.702	0.702 (0.323-1.524)	0.371
Occupation						
Low risk	68 (46.9)	213 (71.7)	Ref		Ref	
High risk	77 (53.1)	84 (28.3)	2.81 (1.90-4.337)	<0.01	2.821 (1.384-5.749)	0.004
Duration of occupation (high risk)						
1-10 years	3 (3.9)	5 (6)	Ref			
11-20 years	12 (15.6)	19 (22.6)	0.343 (0.074-1.588)	0.17	Not adjusted	
21-30 years	27 (35.1)	40 (47.6)	0.361 (0.146-0.895)	0.028		
>30 years	35 (45.5)	20 (23.8)	0.386 (0.185-0.804)	0.011		
Use of protective equipment						
Yes	9 (11.7)	21 (25)	0.39 (0.19-0.93)	0.03	Not adjusted	
No	68 (88.3)	63 (75)	Ref			
Duration of biomass use (hours/day)						
<5 hours	47 (40.2)	120 (54.3)	Ref		Ref	
>5 hours	70 (59.8)	101 (45.7)	4.69 (2.69-8.15)	<0.01	3.214 (1.612-6.407)	0.001
Ventilation in biomass place						
Indoor ventilated	35 (29.9)	144 (65.2)	0.57 (0.32-1.03)	0.063	0.7 (0.321-1.522)	0.304
Indoor not adequately ventilated	55 (47.0)	13 (5.9)	10.03 (4.72-21.30)	<0.01	8.085 (3.064-21.373)	<0.001
Outdoor	27 (23.1)	64 (29.0)	Ref		Ref	
Industries/factories around <3 km of the residential area						
Yes	8 (61.5)	137 (31.9)	3.41 (1.09-10.61)	0.04	3.5 (0.703-17.121)	0.140
No	5 (38.5)	292 (68.1)	Ref		Ref	
Consumption of vegetables						
<2 times/week	46 (31.7)	32 (10.8)	Ref		Ref	0.058
3-5 times/week	90 (62.1)	141 (46.5)	0.44 (0.26-0.74)	0.002	0.458 (0.205-1.026)	0.001
>5 times/week	9 (6.2)	124 (41.8)	0.05 (0.02-0.11)	<0.001	0.758 (0.025-0.247)	0.001
Body mass index						
Healthy weight	71 (49)	171 (57.6)	Ref		Ref	
Low BMI	65 (44.8)	53 (17.8)	2.95 (2.31-3.76)	<0.001	2.336 (1.119-4.879)	0.002
Overweight and obese	9 (6.2)	73 (24.6)	0.332 (0.17-0.54)	0.001	0.325 (0.114-0.923)	0.025
Type of lung disease						
Pulmonary TB	23 (15.9)	19 (6.4)	2.514 (1.311-4.833)	0.006	1.349 (0.457-3.984)	0.588
Bronchiectasis	2 (1.4)	3 (1)	1.386 (0.228-8.427)	0.723	1.359 (0.102-18.178)	0.816
Asthma	8 (5.5)	47 (15.8)	0.353 (0.161-0.77)	0.010	0.434 (0.150-1.253)	0.123
Interstitial lung disease	5 (3.4)	5 (1.7)	2.079 (0.589-7.346)	0.256	3.058 (0.587-15.939)	0.185
COPD	6 (4.1)	13 (4.4)	0.959 (0.750-1.042)	0.395	0.692 (0.139-3.443)	0.653
No pre-existing lung disease	101 (69.7)	210 (70.7)	Ref		Ref	
Family history of lung cancer						
Yes	9 (6.2)	6 (2.0)	3.21 (1.12-9.19)	0.036	3.31 (0.703-15.615)	0.130
No	136 (93.8)	291 (98.0)	Ref		Ref	

Examination of participant characteristics revealed that age, gender, state, district-wise distribution, and urban/rural residence did not significantly impact lung cancer outcomes (all p>0.05). The middle class had significantly lower odds of developing lung cancer (p-value 0.025, AOR 0.355, 95% CI 0.143-0.881).

In the occupational factors, high-risk jobs had a significant risk of lung cancer (p<0.001, AOR 2.81, 95% CI 1.90-4.337). Individuals working 11-20 years and 21-30 years had significantly lower odds of developing lung cancer compared to those working over 30 years (p=0.02, AOR 0.361, 95% CI 0.146-0.895 and p=0.01, AOR 0.386, 95% CI 0.185-0.804), respectively. The use of respiratory protective equipment was associated with reduced odds of lung cancer (p<0.01, AOR 0.39, 95% CI 0.19-0.93).

Biomass fuel usage was not significantly associated with lung cancer (p=0.14). However, the use of indoor biomass without proper ventilation was associated with significantly higher odds of developing lung cancer (p=0.001, AOR 8.085, 95% CI 3.064-21.373). Those who used biomass for >5 hours/day had higher odds (p<0.001 AOR 3.214 95% CI 1.612-6.407) of developing lung cancer in comparison to those who were using it for <5 hours/day. Using a kerosene stove was not associated with lung cancer (p 0.076).

Living within 3 km of heavy industries was associated with increased odds of lung cancer (p=0.025, COR 3.41, 95% CI 2.34-2.47), although this association lost significance after adjusting for other variables (p=0.140, AOR 3.5, 95% CI 0.703-17.121). Among cases, 107/145 (73.8%) and controls 217/442 (73.1%) were exposed to moderate levels of PM 2.5 (12.1-35.4 mcg/m^3^) for the year preceding their diagnosis. 113/145 (77.9%) of cases and 240/297 (80.8%) of controls had an AQI<100 for the year preceding their diagnosis of lung cancer. Both these variables had no association with lung cancer risk.

Vegetable consumption demonstrated a protective association (p=0.004, AOR 0.758, 95% CI 0.025-0.247) only for those consuming vegetables five times a week. Among the cases, 65/145 (44.8%) had low BMI, 71/145 (48.9%) had normal BMI, and 9/145 (6.2%) were overweight and obese. Among the controls, 53/297 (17.8%) had low BMI, 171/297 (57.6%) had normal BMI, 58/145 (19.5%) were overweight, and 12/297 (5.1%) were obese. BMI was associated with lung cancer outcomes, with lower BMI associated with higher odds of lung cancer (p<0.001, OR 2.95, 95% CI 2.31-3.76). Association with lung cancer remained significant for both the groups, i.e., low BMI and overweight/obese, with the AOR being 0.325 (95% CI, 0.114-0.923) and 2.336 (95% CI, 1.119-4.879), respectively.

Pre-existing lung diseases, like pulmonary tuberculosis and bronchial asthma, showed significant associations with lung cancer (p<0.01, COR 2.514, 95% CI 1.98-3.07 and p<0.01, OR 0.353, 95% CI 0.161-0.77, respectively). Among cases, 23/145 (52.3%), 2/145 (4.5%), 8/145 (18.2%), 5/145 (11.4%), 6/145 (13.6%) had old pulmonary tuberculosis, bronchiectasis, asthma, interstitial lung disease, and non-smoker chronic obstructive pulmonary disease (COPD), respectively.

Family history, though associated with increased odds of lung cancer on bivariate analysis (p 0.036 COR 3.12 95% CI 1.12-9.19), the association was not significant in multivariate analysis (p>0.05). Exposure to environmental tobacco smoke (ETS), smokeless tobacco, alcohol consumption, and oral contraceptive use in females did not show significant associations with lung cancer in this study (all p-values>0.05). Regarding the histological subtypes, adenocarcinoma constituted 92.4% of lung cancer. EGFR mutations were the most common at 55.2%, followed by ALK mutations at 11.0%. The Eastern Cooperative Oncology Group (ECOG) performance status revealed a significant proportion presenting at stages 3 and 4. Patients with lung cancer were separated into two groups, i.e., those with lung cancer with driver mutation and lung cancer without driver mutation. There were 97/145 (66.9%) patients positive for driver mutations and 48/145 patients (33.1%) patients negative for the driver mutations assessed. Except for the previous history of lung disease (p 0.014), none of the variables studied were found to have a significant association with the studied driver mutation (Table 3).

**Table 3:** Association between studied variables and common driver mutations SES: Socio-economic status; AQI: Air Quality Index; ETS: Environmental tobacco smoke; OCP: Oral contraceptive pill

Variable	Mutation (n=97)	No mutation (n=48)	Chi-square test, p-value
Age (in years)			
- 20-31	5 (5.2%)	1 (2.1%)	0.582, not significant
- 31-40	6 (6.2%)	2 (4.2%)	
- 41-50	24 (24.7%)	7 (14.6%)	
- 51-60	34 (35.1%)	20 (41.7%)	
- 61-70	20 (28.9%)	12 (25%)	
- 71-80	8 (8.2%)	6 (12.5%)	
Gender			
- Male	54 (55.7%)	32 (66.7%)	0.205, not significant
- Female	43 (44.3%)	16 (33.3%)	
Address			
- Odisha	59 (60.8%)	30 (62.5%)	0.845, not significant
- West Bengal	38 (39.2%)	18 (37.5%)	
District			
- Central Odisha	48 (49.5%)	48 (49.5%)	0.671, not significant
- Southern Odisha	7 (7.2%)	7 (7.2%)	
- Northern Odisha	4 (4.1%)	4 (4.1%)	
- Medinipur East	25 (25.8%)	25 (25.8%)	
- Medinipur West	13 (13.4%)	13 (13.4%)	
SES			
- Upper and upper middle	33 (34%)	17 (35.4%)	0.950, not significant
- Middle	19 (19.6%)	10 (20.8%)	
- Lower and lower middle	45 (46.4%)	21 (43.8%)	
Occupation risk			
- Low risk	46 (47.4%)	22 (45.8%)	0.228, not significant
- High risk	51 (52.6%)	26 (54.2%)	
Protective gear			
- Used	6 (11.8%)	3 (11.5%)	0.977, not significant
- Not used	45 (88.2%)	23 (88.5%)	
Duration of work in high-risk (years)			
- 1-10	2 (3.9%)	1 (3.8%)	0.909, not significant
- 11-20	7 (13.7%)	5 (19.2%)	
- 21-30	19 (37.3%)	8 (30.8%)	
- 31-40	23 (45.1%)	12 (46.2%)	
Locality			
- Urban	18 (18.6%)	7 (14.6%)	0.551, not significant
- Rural	79 (81.4%)	41 (85.4%)	
Industries			
- Yes	7 (7.2%)	1 (2.1%)	0.203, not significant
- No	90 (92.8%)	47 (97.9%)	
PM 2.5			
- Good	7 (7.2%)	1 (2.1%)	0.378, not significant
- Moderate	69 (71.1%)	38 (79.2%)	
- Unhealthy	21 (21.6%)	9 (18.8%)	
AQI grade			
- Good	75 (77.3%)	38 (79.2%)	0.801, not significant
- Moderate	22 (22.7%)	10 (20.8%)	
Biomass fuel			
- Used	77 (79.4%)	40 (83.3%)	0.571, not significant
- Not Used	20 (20.6%)	8 (16.7%)	
Type of fuel			
- Agricultural residue	38 (49.4%)	22 (55%)	0.752, not significant
- Animal residue	13 (16.9%)	6 (15%)	
- Forest residue	21 (27.3%)	8 (20%)	
- Solid and sewage waste	5 (6.5%)	4 (10%)	
Duration of biomass use/day (hours)			
- <5 hours	29 (37.7%)	18 (45%)	0.284, not significant
- >5 hours	48 (18.2%)	22 (55%)	
Ventilation			
- Yes	24 (31.2%)	11 (27.5%)	0.213, not significant
- No	39 (50.6%)	16 (40%)	
- Outdoor	14 (18.2%)	13 (32.5%)	
Kerosene stoves			
- Yes	15 (15.5%)	4 (8.3%)	0.231, not significant
- No	82 (84.5%)	44 (91.7%)	
ETS			
- Yes	13 (13.4%)	8 (16.7%)	0.599, not significant
- No	84 (86.6%)	40 (83.3%)	
Smokeless tobacco			
- Yes	15 (15.5%)	7 (14.6%)	0.889, not significant
- No	82 (84.5%)	41 (85.4%)	
Vegetable consumption per week			
- ≤ 2	28 (28.9%)	18 (37.5%)	0.497, not significant
- 3-5	62 (63.9%)	28 (58.3%)	
- >5	7 (7.2%)	2 (4.2%)	
BMI			
- Normal	47 (48.5%)	24 (50%)	0.774, not significant
- Low BMI	43 (44.3%)	22 (45.8%)	
- Overweight and obese	7 (7.2%)	2 (4.2%)	
Alcohol consumption			
- Yes	9 (9.3%)	3 (6.3%)	0.533, not significant
- No	88 (90.7%)	45 (93.8%)	
Lung disease			
- Yes	23 (23.7%)	21 (43.8%)	0.014, significant
- No	74 (76.3%)	27 (56.3%)	
Family history			
- Yes	5 (5.2%)	4 (8.3%)	0.455, not significant
- No	92 (94.8%)	44 (91.7%)	
OCP			
- Yes	19 (44.2%)	3 (18.8%)	0.072, not significant
- No	24 (55.8%)	13 (81.3%)	

## Discussion

The study investigated the association between age, sex, occupation, and environmental factors and lung cancer risk among non-smokers. High-risk occupations, exposure to indoor air pollutants, a low BMI, and a history of prior lung diseases were some of the risk factors identified as having a positive association with lung cancer. Belonging to the middle-class strata, higher BMI status, use of respiratory protective equipment by the high-risk groups, and consuming vegetables in the diet >5 times per week were found to offer protection from lung cancer.

Patients in the age group of 50-70 accounted for the majority of the lung cancer population. This was consistent with study findings by Lo et al. done to assess lung cancer risk factors in never smokers [12]. Males accounted for 59.3% of lung cancer diagnoses in non-smokers. Occupational factors, notably high-risk occupations and potential underreporting of smoking status were proposed as contributors to the higher proportion of male cases. Research indicates that between 15% and 20% of self-declared non-smokers had cotinine levels in bodily fluids within a range commonly linked to current smoking [13].

The study revealed that most of the lung cancer cases were hailing from central Odisha similar to the findings in a retrospective study conducted by Chatterjee et al. [14] describing regional cancer distribution in Odisha. This finding suggests environmental risk factors as a potential cause for such an occurrence. The middle class had a significantly reduced risk of acquiring lung cancer however there were no comparable studies in the literature that found a similar finding.

The majority of cases and controls were exposed to moderate PM 2.5 levels (12.1-35.4 mcg/m³), and it had no significant association with the risk of lung cancer. The reliance of our study on average PM 2.5 and AQI levels may have oversimplified the exposure assessment. Also, the study acknowledges the need for a follow-up model, emphasizing the importance of prospective cohort studies with extended follow-up periods to assess lung cancer risk more accurately.

In the current study, the use of biomass alone had no significant association with lung cancer risk, but the location of the biomass room along with its ventilation did indicate significant correlations with lung cancer risk. A similar pattern was found in a prospective cohort study by Kim et al. that coal use alone was not significantly associated, however ever using coal with poor ventilation showed a significant association with lung cancer [15]. In our study, individuals exposed to biomass fumes for more than 5 hours had a higher risk of lung cancer, highlighting the association between prolonged exposure to carcinogens like PM 2.5 and increased lung cancer risks [16].

Similar to the study by Consonni et al., our study highlighted that being employed in a high-risk job had significantly higher odds of developing lung cancer [17].

In our study low BMI was significantly associated with lung cancer risk, though it was unclear if low BMI is a result of underlying malignancy or a constitutively low BMI, warranting a follow-up study to understand the association with lung cancer.

We found that increased frequency of vegetable consumption was associated with reduced lung cancer risk similar to a follow-up study by London et al. that discovered certain vegetable intake was linked to a lower risk of lung cancer [18].

Heterogeneity in bronchiectasis etiology and its severity could make it difficult to find a clear association between lung cancer and bronchiectasis. Unlike in other studies, interstitial lung disease and COPD did not have a significant association with lung cancer risk [19,20]. Asthma was protective for lung cancer; however, the association held significance in bivariate regression analysis but failed to hold significance in the multivariate regression analysis. A retrospective cohort analysis suggested that regular usage of inhaled corticosteroids could aid in the prevention of lung cancer [21]. In a pooled analysis of previous lung diseases and lung cancer risk studies, never smokers with old pulmonary tuberculosis had an RR=1.50, (95% CI: 1.03, 2.19) of developing lung cancer [22].

The usage of oral contraceptive pills (OCPs), smokeless tobacco, and ETS was examined in relation to the risk of lung cancer. In the study, none of these variables showed any significant association with the risk of lung cancer. Smokeless tobacco, although showed a significant association with oropharyngeal and prostate cancer in a systematic review by Lee et al. did not increase the risk of lung cancer [23]. The use of OCPs also yielded conflicting results, with a meta-analysis by Wu et al. finding no significant association, while a case-control study by Kreuzer et al. suggested a reduced risk [24,25]. Additionally, exposure to ETS did not demonstrate a significant association with lung cancer risk in our study, consistent with findings from the study by Boffetta et al. [26].

The major histological subtype in 92.4% of the patients was adenocarcinoma. This was consistent with the study by Couraud et al. which found that the prevalence of adenocarcinoma in never smokers ranged from 85% to 87.9% [27]. Nonetheless, a disparity in the ECOG stage distribution was seen, with 55% of the study patients in stages 3 and 4, as opposed to the study by Kawaguchi et al. where 45% of lung cancer patients presented at ECOG stage 1 [28].

The study's secondary objective investigated the link between driver mutation status and determinants of lung cancer in non-smokers. Driver mutation determination was not significantly influenced by any of the studied variables, except for the pre-existing lung disease. According to research by Oxnard et al., germline carriers with familial lung cancer had a significant frequency of EGFR-driver lung adenocarcinomas [29]. A study by Paris et al. discovered that patients who had been exposed to asbestos had a lower frequency of EGFR mutations than patients who had not. However, no significant correlations were found between the other mutations that were studied and various carcinogens, such as silica, polycyclic aromatic hydrocarbons, diesel exhaust fumes, and chrome paint [30].

This study is not a population-based study but a single-center study covering the country’s eastern region. Due to its moderate sample size, the study might have faced limitations in its statistical power as it tried to identify the risk factors for lung cancer in non-smokers. Inter-relationships between the studied variables because of collinearity could have affected the final estimates of the multivariate analysis.

## Conclusions

Several risk factors, both positively and negatively associated with the risk of lung cancer, were identified in this case-control study. The frequency of various histological subtypes and the associated driver mutations reflect the disease heterogeneity in the studied population. These findings provide important information for future studies and public health initiatives trying to prevent lung cancer in non-smokers.
